# The epidemiology of invasive disease due to *Haemophilus influenzae* serotype a in the Canadian North from 2000 to 2010

**DOI:** 10.3402/ijch.v72i0.21142

**Published:** 2013-08-05

**Authors:** Jenny L. Rotondo, Lindsey Sherrard, Melissa Helferty, Raymond Tsang, Shalini Desai

**Affiliations:** 1 Centre for Immunization and Respiratory Infectious Diseases, Public Health Agency of Canada, Ottawa, Ontario, Canada; 2 Syphilis Diagnostics and Vaccine Preventable Bacterial Diseases, National Microbiology Laboratory, Public Health Agency of Canada, Ottawa, Ontario, Canada

**Keywords:** *Haemophilus influenzae*, Hia, emerging infections, infectious diseases, invasive bacterial diseases, Arctic, Northern Canada, Aboriginal, surveillance

## Abstract

**Introduction:**

The International Circumpolar Surveillance (ICS) project is a population-based surveillance network. Since 2000, Canada has participated in the ICS Invasive Bacterial Disease Working Group's surveillance of invasive disease due to *Haemophilus influenzae* (Hi).

**Methods:**

A standardized case report form containing demographic and clinical information was completed for all reported Hi cases in the study regions. Isolates were sent to a reference laboratory for confirmation and serotyping. Analysis was conducted on all Hi serotype a (Hia) cases reported from 2000 to 2010. The northern Canadian population was estimated using Statistics Canada information.

**Results:**

Of the 130 Hi cases reported from 2000 to 2010, 72 (56% of cases with serotype information) were due to Hia. The number of Hia cases reported each year ranged from 2 in 2008 to 13 in 2010. The average Hia incidence over the 11 years was 4.6 cases per 100,000 population per year. The majority of Hia occurred in infants less than 2 years of age (73% of cases). This age group had an average annual incidence of 87.5 cases per 100,000 population. Among cases for which ethnicity was indicated, 91% of Hia cases reported Aboriginal status with the average incidence being 6.9 cases per 100,000 population per year. The most common clinical presentation was meningitis (reported in 37% of cases), followed by bacteraemia (34%) and pneumonia (27%). More than 90% of cases were hospitalized, and there were 4 deaths, resulting in a case fatality ratio of 5.6%.

**Conclusion:**

In the last decade, Hia has become an important cause of morbidity and mortality in the Canadian North. More detailed surveillance information from a national perspective is needed. Further work on vaccine development should be encouraged.

*Haemophilus influenzae* (Hi) is a gram negative coccobacillus that can cause disease in humans, both invasive (e.g. meningitis and septicaemia) and non-invasive (e.g. otitis media). Six encapsulated serotypes (a–f) have been identified, although unencapsulated serotypes are collectively referred to as non-typeable. Historically, invasive disease due to Hi has been primarily caused by serotype b (Hib), especially among those 4 years of age and under (1). Hib is a vaccine preventable disease, with the first vaccine being introduced in Canada in 1986. The *H. influenzae* serotype b conjugate vaccine became a routine part of the childhood immunization schedule for all Canadian children in 1992. Since its introduction, the incidence of Hib has drastically decreased; however, the emergence of invasive disease due to non-b serotypes has been documented both nationally (2, 3) and internationally (4–6). Furthermore, there have been increasing reports of severe disease associated with non-b serotypes (7). At present, there are no available vaccines that offer protection against non-b serotypes.

The International Circumpolar Surveillance (ICS) network was created in 1999 to provide a means of assessing, monitoring, and analyzing population-based rates of infectious diseases in the Arctic Region (8). Through this network, surveillance is conducted on invasive bacterial diseases in northern Canada. Previous research from the ICS network indicated that serotype a was the most frequently reported serotype among Hi cases in the North American Arctic region (9). The emergence of serotype a as a significant cause of invasive disease has also been observed among the Canadian paediatric population (10–12). Given the occurrence and severity of Hia in the Arctic regions (9), continued surveillance of this invasive disease is necessary. The objective of this research article is to review and present the epidemiology of Hia in northern Canada from 2000 to 2010.

## Methods

In Canada, 6 northern Canadian regions participate in the ICS network, including the Northwest Territories, the Yukon, Nunavut, the Québec Cree and Nunivak regions, and northern Labrador. For a detailed description of the ICS network, see Parkinson, Bruce, and Zulz (8). Using the ICS network of public health personnel and laboratories, all laboratory-confirmed cases of Hi from 2000 to 2010 with residence in any of the participating regions were identified and included in this study. Laboratory confirmation included isolation of *H. influenzae* from a normally sterile site or an epiglottic sample, as previously described (8).

A series of laboratories, including 2 reference laboratories, contribute to the surveillance network. Isolates from confirmed cases were sent to the Provincial Laboratory for Public Health in Alberta and the Laboratoire de santé publique du Québec in Québec for characterization and antimicrobial susceptibility testing. Serotyping was done by both bacterial agglutination with commercial antisera and polymerase chain reaction (PCR) (10). Both laboratories participate in ongoing quality control testing (13). Epidemiological information was collected by a communicable disease officer in the participating regions using a standardized case report form that includes data such as demographic information, risk factors for disease, relevant immunization status, outcome, and clinical manifestations. The laboratory and epidemiological data were forwarded to the Centre for Immunization and Respiratory Infectious Diseases (CIRID) at the Public Health Agency of Canada (PHAC) for collation and analysis. Laboratory and epidemiological data were entered into an Access 2003 database and verified by each participating region during annual data audits.

Population estimates were obtained using Statistics Canada's annual census subdivision and territorial July 1 estimates. These estimates were obtained on an annual basis. Aboriginal population estimates were developed for each year using data from the 2006 Canadian Census. Excel 2010 and SAS version 9.1 were used for the analysis.

## Results

**Fig. 1:**
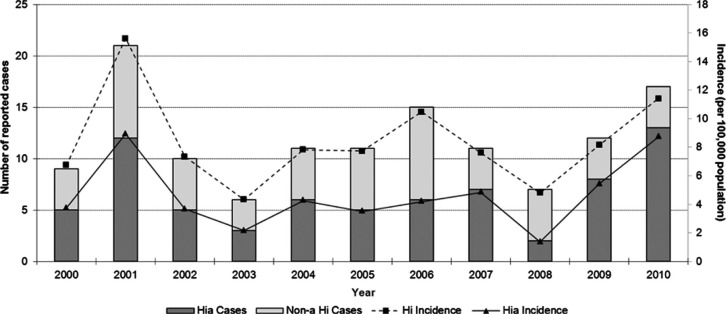
Reported cases and incidence (per 100,000 population) of invasive disease due to *Haemophilus influenzae* in northern Canada by year, 2000 to 2010.

Between 2000 and 2010, a total of 130 Hi cases were reported in participating Canadian ICS regions, with the number reported ranging from 6 to 21 cases per year. Of the 130 cases, 128 (99%) had serotype information and 72 of these (56%) were Hia. The average annual incidence rate of Hi and Hia during this period was 8.4 and 4.6 cases per 100,000 population per year, respectively. The number and proportion of Hia cases (Fig. 1) fluctuated slightly throughout the surveillance period from a low of 2 cases in 2008 (29%) to a high of 13 cases in 2010 (76%). Each year, the proportion of Hia cases was higher than any other serotype reported, with the exception of 2008.

Invasive illness due to serotype a mainly occurred among the young. Infants less than 2 years of age had the highest incidence of Hia (87.5 cases per 100,000 population) and made up the greatest proportion of Hia cases (73%). The age group with the second highest average annual incidence were youth 2 to 19 years of age (2.8 cases per 100,000 population). This age group made up on average 18% of cases annually. Adults 20 to 64 years old and 65 years of age and greater both had an average annual incidence of 1.1 cases per 100,000.

Aboriginal persons were disproportionately represented among those affected by Hia. Self-identified Aboriginal status (First Nations, Inuit, or Métis) was documented in 91% of cases with the remaining 9% having no listed ethnicity. No cases were identified as Non-Aboriginal. Within the Aboriginal group, self-identified Inuit people represented the majority of cases.

The clinical presentation of Hia cases varied, with some reporting more than one. Meningitis was the most common clinical manifestation, occurring in 37% of cases. Bacteraemia (34%) and pneumonia (27%) were also commonly reported among cases. Other reported clinical presentations included septic arthritis (10%), cellulitis (9%), osteomyelitis (4%), and empyema (3%). Hospitalizations and deaths reported throughout the surveillance period were used to assess severity of illness. Hospitalization occurred in more than 90% of all Hia cases for which hospitalization data was available (n=62). Four deaths were reported, resulting in an overall case fatality ratio of 5.6%.

## Discussion

From 2000 to 2010, serotype a was the most frequently reported serotype among Hi cases in northern Canada. In addition, serotype a appears to be causing severe disease, especially among infants less than 2 years of age and within the Aboriginal population. In this population, incidence of Hia among infants less than 2 years of age exceeds that of Hib among children under 5 years of age in the general Canadian population during the pre-vaccine time period (14). Previous research from the ICS region reported similar findings because they observed the occurrence of meningitis in young Hia cases (9). Whether the current level of disease occurrence is due to an unmasking of previously undetected Hi non-b cases or to strain replacement associated with the introduction of serotype b vaccine programs is unknown and is difficult to assess because surveillance of Hi non-b strains was not conducted in this region prior to the introduction of serotype b vaccine programs. In Canada, serotype information is not collected on nationally reported Hi cases other than serotype b. Given the heavy burden of disease reported, an expansion of national Hi surveillance activities should be considered.

The population demographics in the ICS region are unique to the rest of Canada in terms of ethnicity and age. Among the participating regions, 57% of people are self-identified Aboriginal (First Nations, Inuit, or Métis) and make up about 7% of the Canadian Aboriginal population. Consistent with previously published literature, these data suggest that North American Aboriginal people are overrepresented among Hia cases (2, 9). In addition, among Canadian paediatric cases from 1996 to 2001, Aboriginal ethnicity was an important risk factor for Hia disease (2). Prior to and following the introduction of Hib conjugate vaccines, a higher incidence of Hib was observed among Aboriginal populations worldwide, as compared to the general population. However, mixed evidence as to the role of genetic (15, 16) or environmental (17, 18) factors requires further investigation. Additionally, the median age in the ICS region is much lower than the rest of Canada; 9% of the population is less than 5 years of age compared to 6% for all of Canada (19). Given the propensity of Hia to manifest among young children, the age composition may also contribute to the high incidence of Hia disease.

The polysaccharide capsule plays a major role in the overall virulence of Hi because it protects the core bacterial components from the lytic activity of complement (20). Of all the non-b serotypes, the polysaccharide capsule of serotype a is the most similar to that of serotype b because both are composed of a neutral sugar, an alcohol (ribitol), and either glucose (in Hia) or ribose (in Hib) linked in a phosphodiester linkage (20). Given these structural similarities, it is not unexpected that serotype a has the potential to cause clinically significant disease. Invasive, virulent disease caused by serotype a has been increasingly documented in the post-Hib vaccination era (21). Because there is currently no vaccine against Hia, laboratory surveillance encompassing serotype information and patient information (including clinical history) is important and could contribute to the prioritization of research into novel vaccine candidates.

Previous studies have identified the partial deletion involving IS*1016-bexA* genes as responsible for enhanced fitness and increased virulence of *H. influenzae*, by allowing for Cap gene amplification (22). This results in enhanced capsular polysaccharide production and increases the likelihood of survival within the host (23). Though historically found in *H. influenzae* serotype b, the IS*1016-bexA* deletion has been identified in *H. influenzae* serotype a strains that caused serious disease associated with poor outcome (24, 25), some cases of which occurred in Canada (26). Although previous investigation failed to identify the IS*1016-bexA* deletion among Hia isolates in the ICS region (9)—therefore deeming it unlikely to be the cause of the high rates of invasive Hia disease in the ICS region—the appearance of this deletion within the ICS Hia population could serve as an indication of a pending increase in serious disease.

### Limitations

Although large in size, the ICS region encompasses a small population; therefore, small case numbers need to be considered when interpreting results because even one case can cause great variation in year-to-year incidence rates. Because most cases of invasive illness require medical attention, we anticipate that the majority of cases would be captured through this system. Nonetheless, some cases may not have been captured by this surveillance system due to a lack of reporting by participating clinics or to an inability to isolate the organism as a result of the administration of antibiotics prior to specimen collection. The surveillance system relies on active data collection in an area of Canada that must transfer critically ill patients, resulting in medical records existing in more than one location for a single patient. As a result, some variables—including risk factors, manifestations, and hospitalization—were not complete for all cases, thereby limiting our understanding of predisposing factors for infection and severity of disease.

Finally, during the 2000 to 2010 time period, a transition towards the use of PCR for serotype confirmation occurred. The effect of transitioning to PCR will be the reduced misclassification of typeable Hi as non-typeable and progressively correcting the underestimation of the proportion of all typeable cases, including Hia. Historic typeable case counts might therefore be an underestimation, whereas non-typeable case counts might be an overestimation.

## Conclusion

In the last decade, Hia has been observed in northern Canada and globally. Populations such as young children and Aboriginal groups have been identified as having a greater burden of illness in northern Canada. Development of a vaccine against serotype a may be beneficial in protecting these at-risk populations. The ICS system is an excellent example of a laboratory and an epidemiological partnership; however, it only portrays a small portion of the overall burden of Hi in Canada. In order to monitor the changing epidemiology of Hi disease in Canada, it would be beneficial if all serotypes were reportable at a national level, not just Hib. Monitoring for known virulence enhancing deletions should also be considered.
